# The single D380 amino acid substitution increases pneumolysin cytotoxicity toward neuronal cells

**DOI:** 10.1016/j.isci.2024.109583

**Published:** 2024-03-27

**Authors:** Simona Serra, Vittorio Iannotti, Margherita Ferrante, Miguel Tofiño-Vian, Joseph Baxendale, Gilad Silberberg, Thomas P. Kohler, Sven Hammerschmidt, Andrew T. Ulijasz, Federico Iovino

**Affiliations:** 1 Department of Neuroscience, Karolinska Institutet, Stockholm, Sweden; 2 Department of Molecular Genetics and Infection Biology, Interfaculty Institute for Genetics and Functional Genomics, Center for Functional Genomics of Microbes, University of Greifswald, Greifswald, Germany; 3 Department of Microbiology and Immunology, Loyola University Chicago, Maywood, IL, USA

**Keywords:** Natural sciences, Biological sciences, Biochemistry, Neuroscience, Molecular neuroscience, Microbiology, Medical Microbiology

## Abstract

Bacterial meningitis, frequently caused by *Streptococcus pneumoniae* (pneumococcus), represents a substantial global health threat leading to long-term neurological disorders. This study focused on the cholesterol-binding toxin pneumolysin (PLY) released by pneumococci, specifically examining clinical isolates from patients with meningitis and comparing them to the PLY-reference *S. pneumoniae* strain D39. Clinical isolates exhibit enhanced PLY release, likely due to a significantly higher expression of the autolysin LytA. Notably, the same single amino acid (aa) D380 substitution in the PLY D4 domain present in all clinical isolates significantly enhances cholesterol binding, pore-forming activity, and cytotoxicity toward SH-SY5Y-derived neuronal cells. Scanning electron microscopy of human neuronal cells and patch clamp electrophysiological recordings on mouse brain slices confirm the enhanced neurotoxicity of the PLY variant carrying the single aa substitution. This study highlights how a single aa modification enormously alters PLY cytotoxic potential, emphasizing the importance of PLY as a major cause of the neurological sequelae associated with pneumococcal meningitis.

## Highlights

• Pneumococcal meningitis isolates release more PLY than the reference strain D39
• The PLY of the clinical isolates has a single aa substitution compared to the D39 PLY
• This single aa substitution significantly enhances PLY pore-forming activity
• Higher pore-forming activity leads to higher cytotoxicity toward neurons

## Graphical abstract

**Figure:**
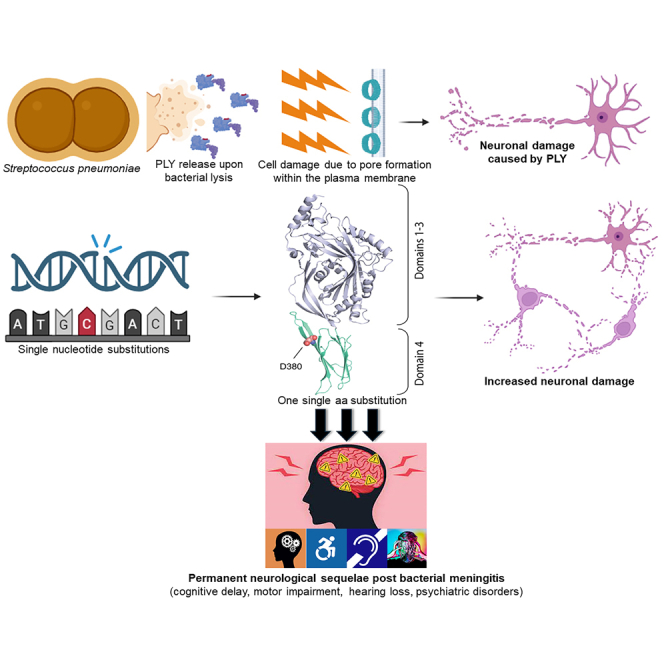

## Introduction

Infections of the brain are a major cause of neurological impairment. The bacterium *Streptococcus pneumoniae* (the pneumococcus) is the main etiological cause of bacterial meningoencephalitis globally.^1^^,^^2^ Pneumolysin (PLY) is a cholesterol-dependent cytolysin (CDC) released by pneumococci and can inflict severe damage to cells through its pore-forming activity and is therefore considered a key virulence factor in pneumococcal pathogenesis.^3^^,^^4^ In fact, it has been shown that PLY can induce neuronal cell death through mitochondrial damage.^5^ More recently, it was reported that PLY allows pneumococci to enter neuronal cells.^6^ Moreover, PLY has been described to bind to exposed β-actin filaments of the neuronal cytoskeleton and, through this interaction, cause cytoskeleton instability culminating to neuronal cell death due to cytoskeleton disruption.^6^ Like other CDCs, PLY contains the highly conserved undecapeptide sequence, also known as the tryptophan-rich loop, and a threonine-leucine amino acid pair, essential for the binding of PLY to membrane cholesterol.^3^ In particular, the domain 4 (D4) was shown to be essential for the initial binding of PLY to membrane cholesterol and such an interaction is the initial step that leads to pore formation on the plasma membrane of the eukaryotic cells targeted by PLY.^7^ Therefore, a disruption in the protein structure of this domain would be predicted to have consequences on PLY activity, and possible patient outcomes.

In this study, we have investigated the genetic, protein, and cytotoxic features of PLY released by clinical strains of *S. pneumoniae* isolated from the blood of patients who died of pneumococcal meningitis.^8^ Gene sequencing analysis and *in silico* protein structure analysis revealed that different pneumococcal meningitis clinical isolates express a variant of PLY which differ from the NCBI-deposited PLY sequence (NCBI Reference Sequence: WP_001284359.1) from a single amino acid substitution in the D4 domain. The new PLY variant from the different clinical isolates, recombinantly produced, had different cytotoxic capabilities, in fact, neurons treated with the new PLY variant from the pneumococcal clinical isolates exhibited higher neuronal cell death. Taken together, these results demonstrate that, even though PLY is highly conserved,^9^ different clinical isolates of *S. pneumoniae* express a variant of PLY which, with just one single amino acid substitution, causes more severe cell damage. Furthermore, scanning electron microscopy analysis revealed that not only more, but also bigger pores were formed on the neuronal plasma membrane when neurons were treated with the PLY from the pneumococcal clinical isolates containing the critical D4 domain mutation, therefore proving that the higher cytotoxicity of the new PLY variant is due to a higher pore-forming activity. Importantly, the neuronal cytotoxicity was not limited to isolated evaluations using only *in vitro* cellular models; in fact, through patch clamp electrophysiological recordings using mouse brain slices, we confirmed that neuronal electrophysiological status is more robustly and quickly impaired when neurons are exposed to the PLY of the clinical isolates than those exposed to the PLY of the reference strain D39. Here, we suggest that the enhanced toxic power observed in the pneumococcal meningitis clinical isolates is a direct consequence of this substitution in the D4 domain which would ultimately be predicted to alter the structural integrity of the D4 domain altering the capability of D4 to anchor to the cell membrane and affecting the strengthening or weakening of the formation of pores.

PLY can be regarded as one of the most dangerous weapons of the pneumococcus. In fact, while antibiotics or antimicrobials can eliminate bacteria, PLY constitutes a singular and distinct danger: upon bacterial lysis induced by potent antimicrobials, such as β-lactam antibiotics, PLY is released in the external environment and is therefore capable of damaging cells and tissues even if the infection is successfully treated.^10^ Permanent neurological sequelae, due to neuronal damage caused by the infection, represent a major burden of bacterial meningitis globally.^11^^,^^12^^,^^13^^,^^14^ Our group and previous authors have previously elucidated the important role of PLY in inducing neuronal cell death upon interaction with neuronal plasma membrane.^4^^,^^5^^,^^6^ Our new findings shed light onto the important concept that one single aa substitution can dramatically increase the cytotoxic power of PLY toward neurons, and therefore, raises awareness on, firstly, the use of bacteriostatic antimicrobials for the treatment of pneumococcal infections and, secondly, the need of new therapeutic strategies able to eradicate the infection and, simultaneously, neutralize the toxic effect of PLY.

## Results

### Most of the pneumolysin produced by S. pneumoniae meningitis clinical isolates is released

**Figure 1:**
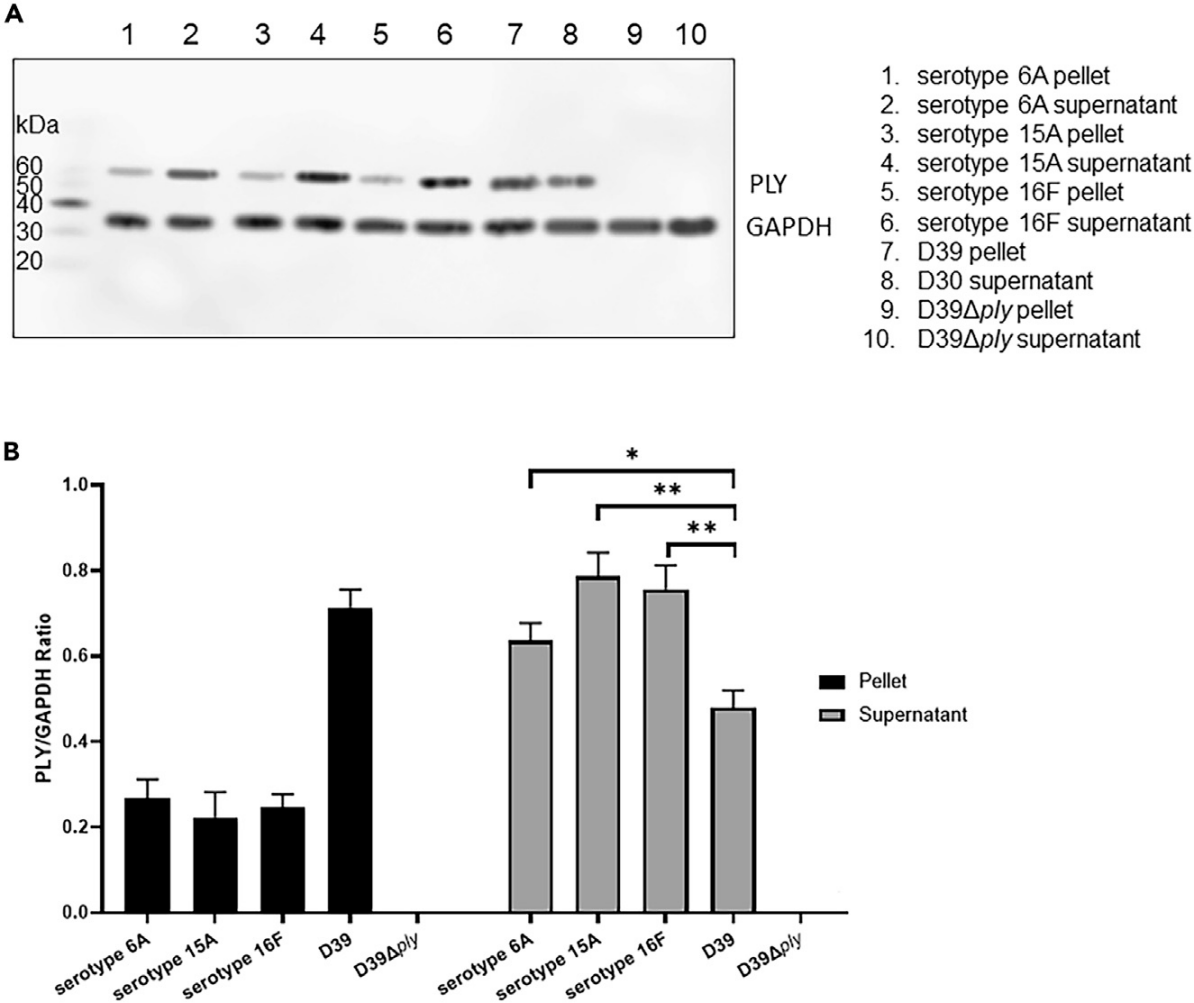
Quantification of PLY released by THY-grown pneumococcal meningitis clinical isolates and D39 reference strain (A) PLY and GAPDH, respectively at approximately 53–55 kDa and 35–37 kDa, detected by western blot analysis in pellet and supernatant from pneumococcal liquid cultures in THY; western blot analysis was repeated three times (n = 3), each time with different cultures of pneumococci, the displayed western blot is representative of three biological replicates. (B) Quantification of PLY (PLY/GAPDH ratio) in pellet (PLY within bacterial cells) and supernatant (released PLY) from the western blot results shown in Figure 1A; columns represent average values and error bars are standard deviations calculated using the PLY/GAPDH values of each strain/isolate among three biological replicates; ∗ = p < 0.05, ∗∗ = p < 0.01 (2-tails ANOVA test was run to assess the presence of differences between the groups, and then a Dunn’s test was applied for pairwise comparisons; p = 0.034 for the comparison between D39 and serotype 6A, p = 0.007 for the comparison between D39 and serotype 15A, p = 0.008 for the comparison between D39 and serotype 16F; Group comparison F = 1.564, T = 7.632, R^2^ = 0.8562, Degree of Freedom = 10).

**Figure 2:**
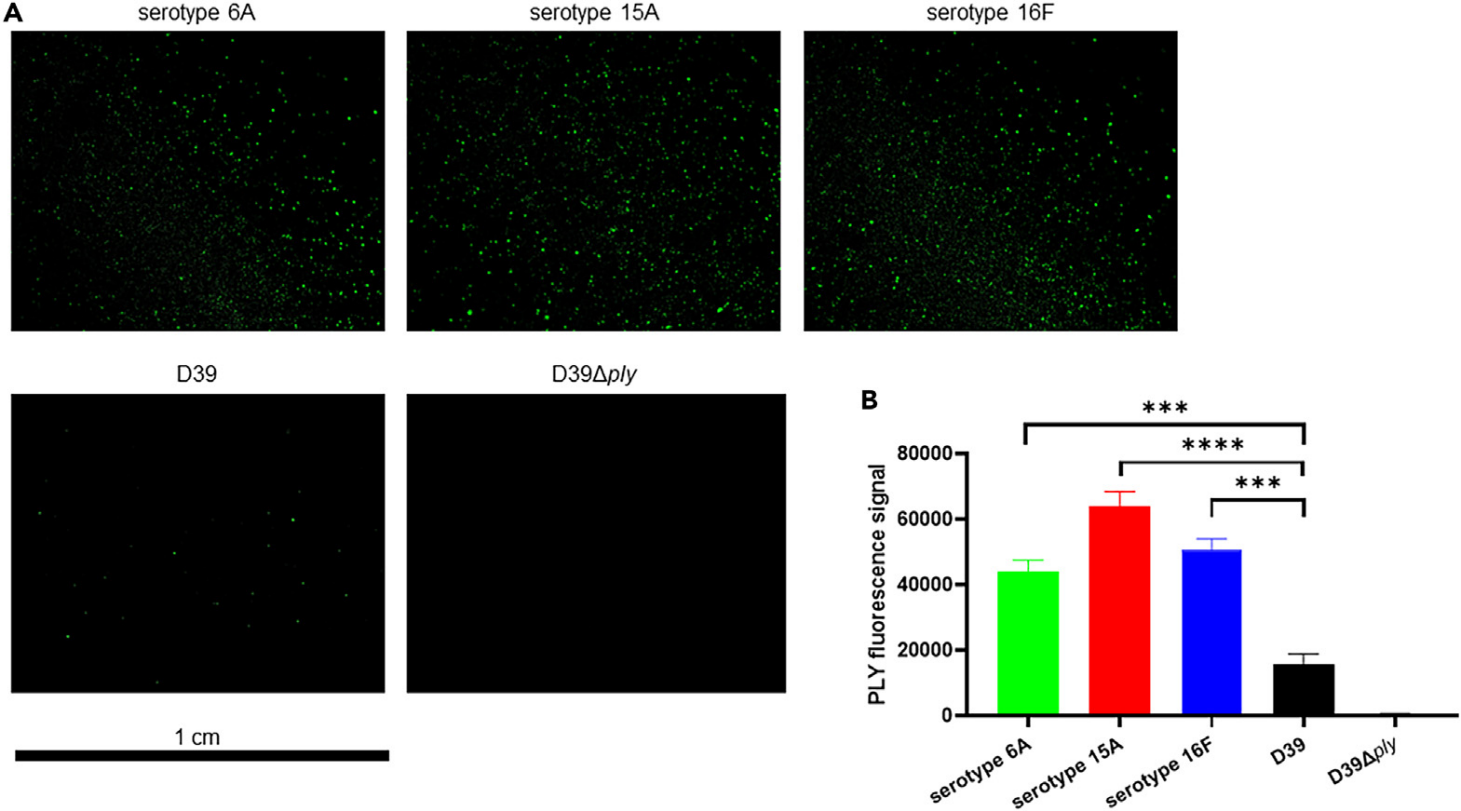
Increased amounts of PLY released by pneumococcal meningitis clinical isolates upon growth in human blood (A) Detection of PLY signal (green) by immunofluorescence microscopy using serum samples from pneumococcal cultures in whole human blood; each image is representative of 15 images taken per each clinical isolate/strain/biological replicate (n = 45). (B) Quantification of the area, measured by ImageJ, occupied by PLY fluorescence signal in each image/field of view (each field of view displayed one dried drop of serum) from the immunofluorescence microscopy analysis shown in Figure 2A; columns represent average values and error bars are standard deviations calculated using the area values of the PLY fluorescence signal in each image/field of view per each strain/isolate; ∗∗∗ = p < 0.001, ∗∗∗∗ = p < 0.0001 (2-tails ANOVA test was run to assess the presence of differences between the groups, and then a Dunn’s test was applied for pairwise comparisons; Group comparison F = 7.732, T = 14.76, R^2^ = 0.5225, Degree of Freedom = 10).

**Figure 3:**
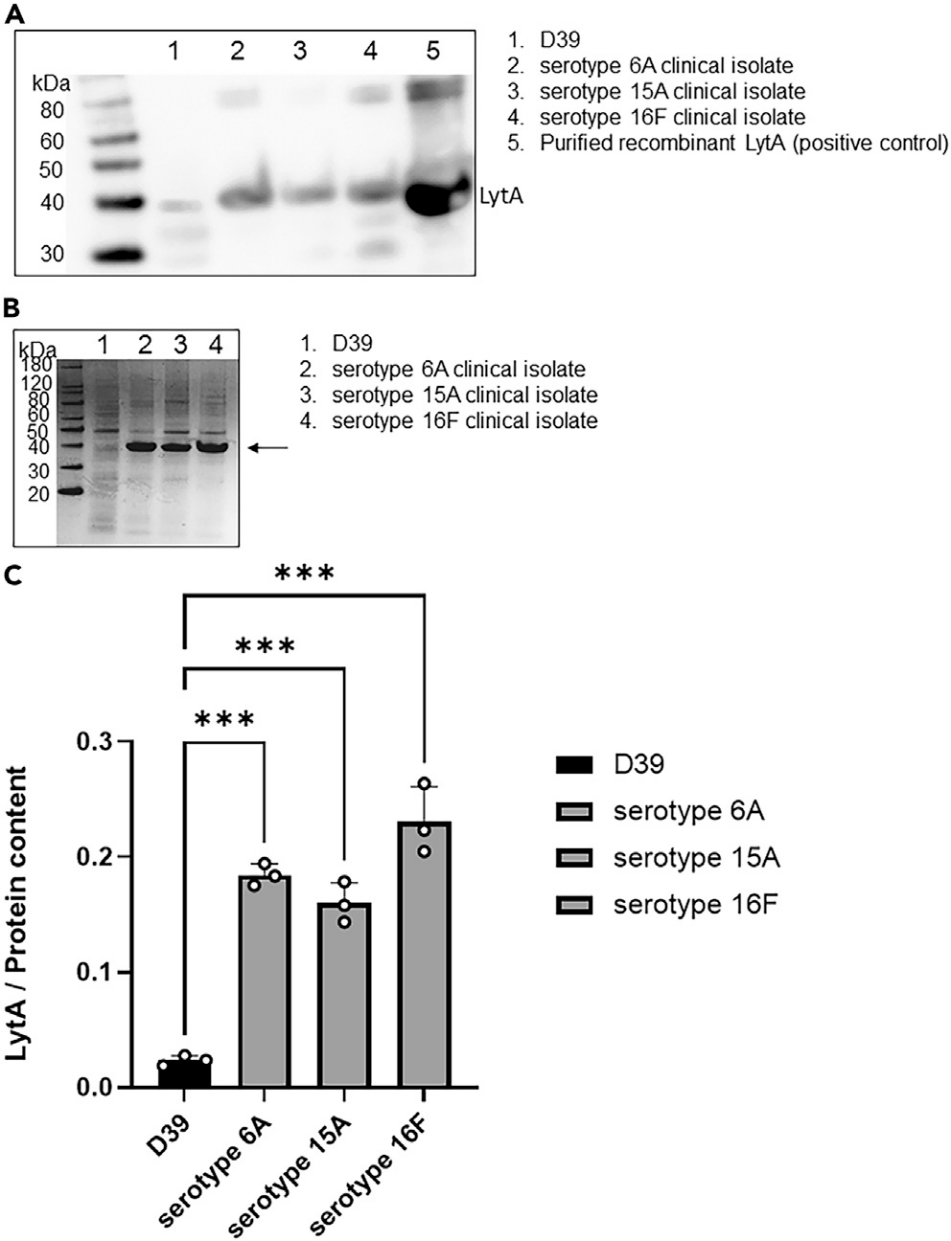
Quantification of LytA expressed by the pneumococcal meningitis clinical isolates and *S. pneumoniae* D39 (A) LytA was detected at approximately 37 kDa by western blot analysis in pneumococcal lysates (pellets); western blot analysis was repeated three times (n = 3), each time with different cultures of pneumococci, the displayed western blot is representative of three biological replicates. (B) Grayscale Coomassie staining of lysates of D39 and the meningitis clinical isolates used as loading control to quantify the expression levels of LytA, the same volume and amount of protein content as the one used for the western blot in Figure 3A was used for the Coomassie staining; the arrow points toward the band where at the same molecular weight LytA was detected; the staining of the whole protein content reveals that, with the same protein quantity, D39 seems to have more proteins within the range of 10 and 40 kDa, whilst the clinical isolates have much more protein content, including LytA, at around 40 kDa. (C) Quantification of LytA (PLY/Protein content ratio) in D39 and the meningitis clinical isolates from the western blot and the Coomassie staining results shown in, respectively, Figures 1A and 1B; columns represent average values and error bars are standard deviations calculated using the LytA/Protein content values of each strain/isolate among the three biological replicates of the LytA western blot (the same Coomassie staining was used for the quantification of LytA in each western blot); ∗∗∗p < 0.001 (2-tails ANOVA test was run to assess the presence of differences between the groups, and then a Dunn’s test was applied for pairwise comparisons; Group comparison F = 1.13, T = 7.212, R^2^ = 0.964, Degree of Freedom = 11).

PLY is a toxin and exerts its pore-forming toxic properties when released by the bacteria, and was recently described that pneumococcal capacity to invade neuronal cells is significantly impaired in the absence of PLY release.^6^ To investigate the pattern of PLY release of different *S. pneumoniae* meningitis clinical isolates, we first assessed pneumococcal growth in the complex medium THY, of three clinical isolated, where all isolates exhibited a growth curve that paralleled that of the laboratory strain D39 (Figure S1). The serotype 2 strain D39 was chosen as a “laboratory strain reference” because it is the referenced strain related to the NCBI *ply* gene (Gene ID: 66806991).^15^ We then analyzed the pattern of PLY release of both THY-grown pneumococcal clinical isolates and the D39 reference strain. Western blot analysis showed that, for all three clinical isolates, most of the PLY produced was found in the supernatant, while the abundance of PLY in the supernatant of strain D39 was lower than in the bacterial pellet (Figures 1A and 1B). To assess whether the same PLY release pattern occurred also in the natural pathological conditions, we first assessed the capability of the clinical isolates to grow in anticoagulated human blood and observed a similar growth rate for all three isolates and D39 (Figure S2). We then analyzed the amount of released PLY in the clinical isolates and D39 upon growth in whole human blood, and immunofluorescence microscopy analysis confirmed that the amount of PLY released, present in the serum, was significantly higher for the three clinical isolates when compared to the D39 reference strain (Figures 2A and 2B). To determine whether, in the clinical isolates, the great abundance of PLY in the released content was due to a higher expression of LytA, the major autolysin in *S. pneumoniae*,^16^ we performed western blot analysis using bacterial lysates of D39 and the meningitis clinical isolates. As a result, all the clinical isolates had a significantly higher expression of LytA than D39 (Figures 3A and 3B), and these data most likely explain the higher propensity of the clinical isolates to release PLY in the external environment.

### Pneumococcal meningitis clinical isolates express a different pneumolysin variant

**Figure 4:**
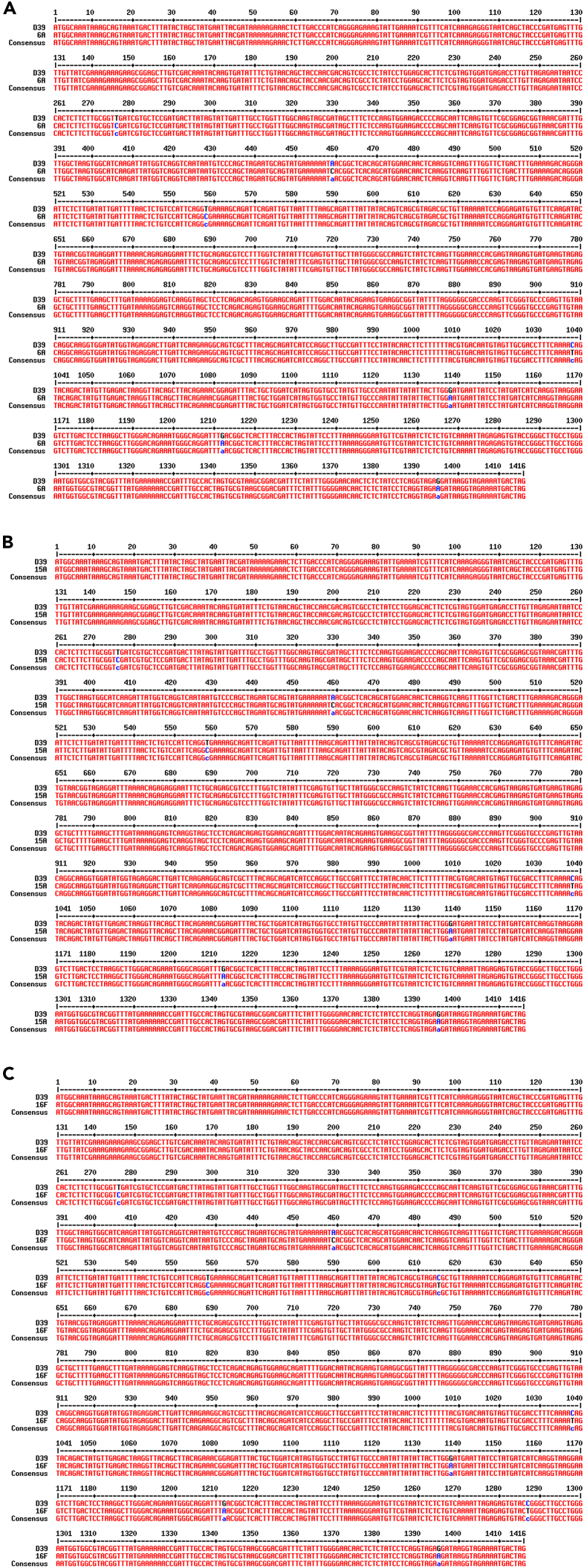
Alignment of *ply* gene sequences from *S. pneumoniae* D39 reference strain and pneumococcal meningitis clinical isolates *Ply* gene sequence of D39 was aligned along with the *ply* gene sequence of the serotype 6A (A), 15A (B) and 16F (C) pneumococcal meningitis clinical isolates; gene sequence alignment was performed using the Multalign online tool, the “Consensus” sequence represents the overlap between the D39 *ply* sequence and the *ply* sequence of the meningitis clinical isolates; nucleotide substitutions are marked in blue.

**Figure 5:**
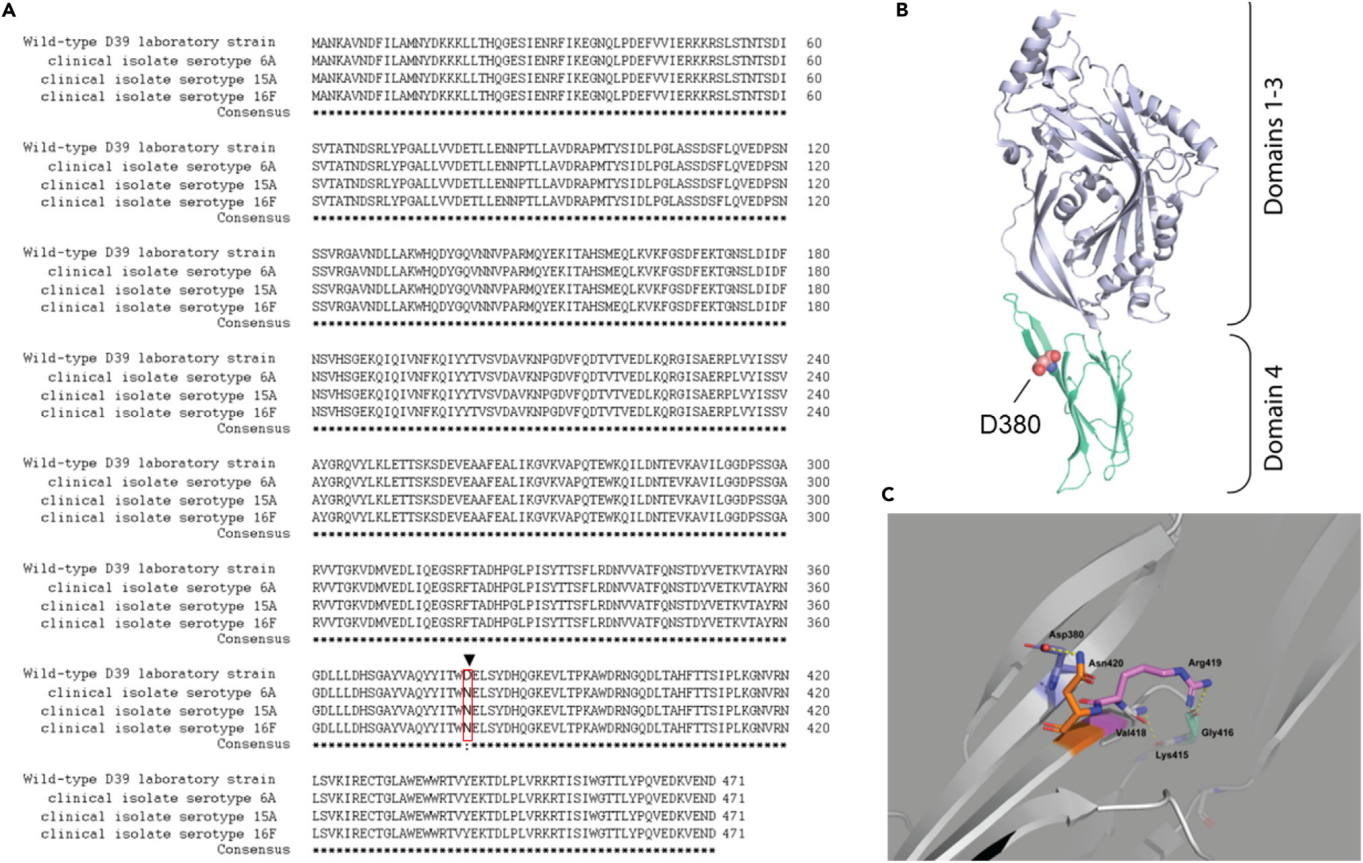
A single amino acid substitution changes the conformation of the D4 domain in the new PLY variant of the pneumococcal meningitis clinical isolates Conservation and structural interpretation of PLY variants. (A) Clustal Omega alignment of the wild-type D39 PLY aa sequence and the three clinical isolates. The critical D380 > N mutation is boxed in red. (B) Cartoon model of the pneumococcal PLY structure (PDB code 5AOD) showing the placement of the Asp380 residue and (C) closeup of its hydrogen bonding with nearby residues in which potential static interactions are shown as dashed yellow lines.

Upon the release in the external host compartment, PLY can exert its cytolytic and cytotoxic effects. To investigate the degree of cytotoxicity toward neurons of the PLY released by the three pneumococcal clinical isolates, we performed *in vitro* infection experiments using human neuronal cells differentiated from SH-SY5Y cells and combined with lactate dehydrogenase (LDH) release assays (to measure cell death). Matching the same amount of PLY release in the supernatant according to western blot analysis, the three clinical isolates showed a significantly higher toxicity toward human SH-SY5Y-derived neurons compared to the toxicity observed for D39 (Figure S3). Even though pneumococci have many virulence factors besides PLY capable of inducing cellular damage, this finding suggests that PLY released from pneumococcal clinical isolates likely differed from the one of the D39. We then analyzed more thoroughly the PLY expressed by the three clinical isolates at both gene and amino acid sequence levels. The *ply* gene of three pneumococcal meningitis clinical isolates was sequenced and aligned with reference D39’s *ply* sequence (NCBI gene ID: 66806991), revealing that the clinical isolates serotypes 6A and 15A differed in seven nucleotides (Figures 4A and 4B) and the clinical isolate 16F nine nucleotides in comparison to the D39 reference sequence (Figure 4C). These nucleotide differences all translated into one amino acid (aa) substitution from the D39’s PLY WP_001284359.1-PLY aa sequence (NCBI Reference Sequence WP_001284359.1). The common switch leads to a single amino acid substitution from aspartate to asparagine (Figure 5A). Notably, this amino acid change occurs in the D4 domain for all three clinical isolates (Figure 5B). When examined within the available PLY atomic structure (PDB code 5AOD; deposited by van Pee, K. and Yildiz, O. https://www.rcsb.org/structure/5AOD), this change in the same rotamer configuration would result in a steric clash (negative - negative) in charges between R group amides of the residues Asp380 and Asp420 and could then affect Arg419 contacts with the Gly416 loop hinge, thereby affecting the conformation of the D4 domain (Figure 5C). Interestingly, according to the NCBI Protein database, the other commonly used *S. pneumoniae* TIGR4/serotype 4 strain shares the same PLY variant of the three clinical isolates (Figure S4). This is not surprising since TIGR4, originally isolated from human blood, is itself a clinical isolate.^17^ Moreover, the ongoing prevalence of serotype 4 in causing pneumococcal meningitis further supports this observation.^18^

### A single amino acid substitution significantly enhances the neuronal cytotoxicity of pneumolysin

**Figure 6:**
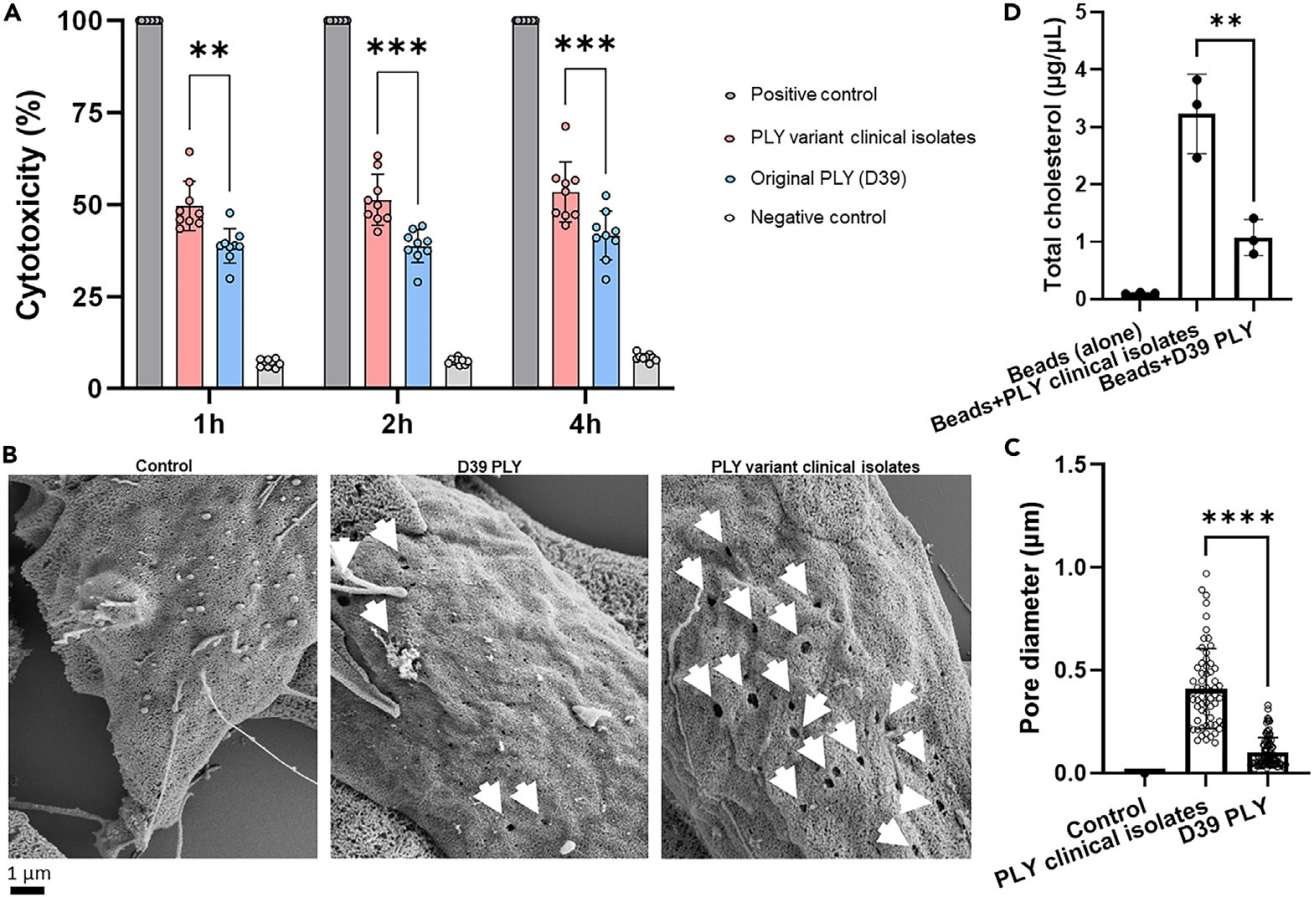
The PLY of the pneumococcal meningitis clinical isolates is more neuronal cytotoxic than the PLY of D39 due to a stronger capability to interact with cholesterol which leads to an enhanced pore-forming activity (A) LDH release assay using human SH-SY5Y-derived neuronal cells treated with the two recombinant PLY variants (original PLY from D39 and the PLY variant from the pneumococcal meningitis clinical isolates) showed the degree of neuronal cell death (% cytotoxicity) caused by the toxin; cells treated with lysis buffer were the “Positive control,” untreated cells were the “Negative control”; three biological replicates (three technical replicates in each biological replicate) were performed (n = 3), columns represent average value, datapoints represent all the technical replicates of all three biological replicates; cytotoxicity of the Positive control was set as 100%; ∗∗ = p < 0.01, ∗∗∗ = p < 0.001 (2-tails ANOVA test was run to assess the presence of differences between the groups, and then a Dunn’s test was applied for pairwise comparisons; p = 0.002 for the comparison between PLY of clinical isolates and D39 PLY at 1h; Group comparison F = 1.581, T = 7.714, R^2^ = 0.9370, Degree of Freedom = 4). (B) Scanning electron microscopy of the cell surface of SH-SY5Y-derived neuronal cells after 30 min of treatment with either the recombinant PLY of D39 (original PLY) or the recombinant PLY of the pneumococcal meningitis clinical isolates (new PLY variant), untreated neuronal cells were used as control; Approximately 120 neuronal cells were images per each experimental condition (untreated as “Control,” treatment with original PLY, treatment with new PLY variant), pores are indicated by white arrows. (C) Measurement of the pore diameter on the plasma membrane of neuronal cells treated with either PLY of clinical isolates of D39 PLY; each datapoint in scatter dot plot represents one pore in each individual image analyzed by SEM (n = 56), columns represent average values and error bars are the standard deviations; all the SEM images in which pores had a regular circle shape were used for this quantification; ∗∗∗∗ = p < 0.0001. (D) Quantification of cholesterol bound to either the recombinant PLY of the meningitis clinical isolates or the PLY of D39, or to the beads alone; bars represent average values, error bars the standard deviations; Each datapoint represents the quantification of cholesterol performed in one biological replicate of the pull-down experiment (three biological replicates in total, n = 3); the amounts of cholesterol bound to either recombinant PLYs or beads alone was adjusted by matching the same amount of PLY bound to the beads according to the Western blot-based quantification of PLY bound to the beads shown in Figures 5A–5C; ∗∗ = p < 0.01 (2-tails ANOVA test was run to assess the presence of differences between the groups, and then a Dunn’s test was applied for pairwise comparisons; p = 0.008 for the comparison between “Beads+PLY clinical isolates” and “Beads+D39 PLY”; Group comparison F = 1.13, T = 4.904, R^2^ = 0.8574, Degree of Freedom = 4).

The two purified recombinant PLY variants, the one released by the three clinical isolates and the one of the reference strains D39, were then used to further investigate their cytotoxic properties toward neuronal cells. SH-SY5Y-derived neurons were treated with identical amounts of the two PLY variants. Notably, LDH assays showed that the PLY variant from the clinical isolates had a significantly higher cytotoxicity compared to PLY of D39 (Figure 6A). Moreover, scanning electron microscopy analysis of SH-SY5Y-derived neurons treated with either the PLY variant of the clinical isolates or PLY of D39 further revealed that the PLY variant from the clinical isolates formed not only more but also bigger pores on neuronal plasma membrane than what observed for PLY of D39 (Figures 6B and 6C). Binding of PLY to membrane cholesterol is a fundamental step in the pore formation process.^19^ A stronger interaction with cholesterol could explain the higher pore-forming capability of the PLY variant of the clinical isolates. Pull-down experiments with Ni-NTA magnetic beads coupled to either His-tag recombinant PLY variants incubated with purified cholesterol showed that the PLY of the clinical isolates was able to bind amounts of cholesterol that were significantly higher than what observed for the PLY of D39 (Figure 6D); importantly, western blot analysis showed that similar quantities of PLY variants were coupled to the beads (Figures S5A–S5C). Overall, these findings demonstrated that the higher cytotoxicity of the new PLY variant is due to a stronger binding to cholesterol which leads to an enhanced pore-forming activity.

**Figure 7:**
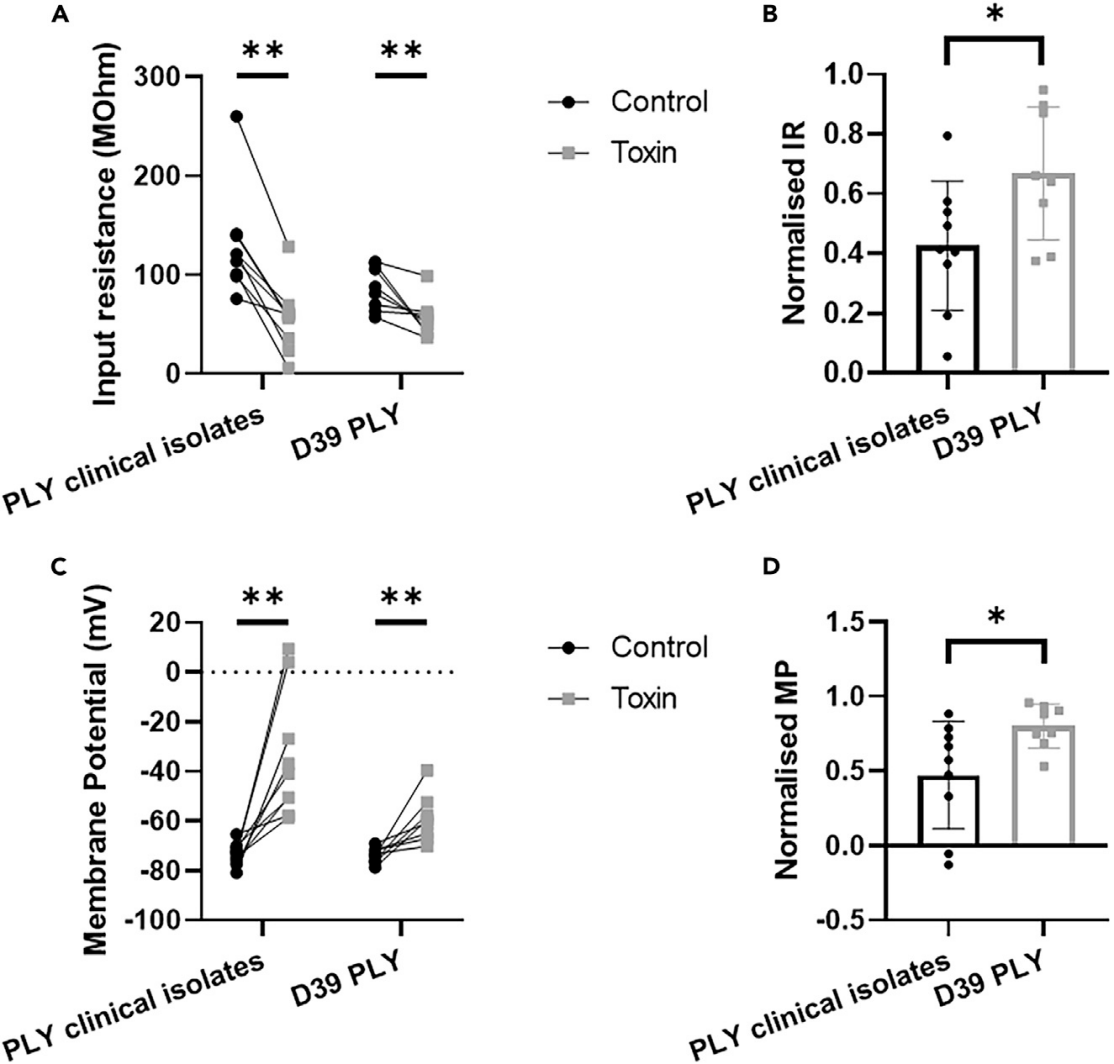
*Ex vivo* whole cell patch clamp electrophysiological recording confirms that the PLY variant of the meningitis clinical isolates functionally impairs neurons quicker and more robustly than the PLY of D39 Whole cell patch clamp recordings were obtained from striatal neurons *ex vivo* and the membrane voltage responses to a series of current steps were acquired before and after the bath application of PLYs. A single current injection step was intermittently applied to the neuron to monitor the health of the neuron (Control). Over time, neurons exposed to either PLY variants showed signs of deteriorating neuronal health when compared to control. (A) Input resistance of recorded neurons before (black) and after (gray) the addition of the toxin; n = 9 nr of neurons in brain slices incubated with ACSF with the addition of PLY of clinical isolates, n = 9 nr of neurons in brain slices incubated with ACSF with the addition of PLY of D39. (B) Change in input resistance (IR) as a proportion of control recordings in the presence of PLY of the meningitis clinical isolates (black) or PLY of D39 (gray) according to the input resistant measurements shown in Figure 7A. (C) Membrane potential (MP) of recorded neurons before (black) and after (gray) the addition of toxin; n = 9 nr of neurons in brain slices incubated with ACSF with the addition of PLY of clinical isolates, n = 9 nr of neurons in brain slices incubated with ACSF with the addition of PLY of D39. (D) Change in membrane potential as a proportion of control recordings in the presence of presence of PLY of the meningitis clinical isolates (black) or PLY of D39 (gray) according to the input resistant measurements shown in Figure 7C. ∗ = p < 0.05, ∗∗ = p < 0.01 (p = 0.037 for the comparison between PLY clinical isolates and D39 PLY concerning normalized IR, p = 0.027 for the comparison between PLY clinical isolates and D39 PLY concerning Normalized MP, p = 0.003 for the comparison of IR and MP before and after PLY clinical isolates, p = 0.007 for the comparison of IR and MP before and after D39 PLY; Data in Figures 6A–6D resulted not in normal distribution and Wilcoxon test was applied with a Degree of Freedom = 9; Data in Figure 6B resulted in normal distribution and unpaired t-test was applied, F = 1.064, T = 2.282, R^2^ = 0.2578, Degree of Freedom = 15).

To further assess the different neuro-cytotoxic capabilities of the two PLY variants beyond the use of *in vitro* cellular models, whole cell patch clamp recordings were obtained from striatal neurons within *ex vivo* mouse brain slices, and the membrane voltage responses to a series of current steps were acquired before and after the bath application of either the PLY variant of the clinical isolates or the PLY of D39. Since the main action of the toxin is to perforate cell membranes, a key measure of functional activity is a reduction in input resistance. Both the clinical isolate PLY and the PLY of D39 decreased the input resistance of recorded neurons (Figure 7A). However, this decrease was larger for neurons exposed to the new PLY variant than the D39 PLY compared to control recordings before the action of the toxin (Figure 7B). These data suggest that, while both toxins perforate neuronal membranes, the PLY of the clinical isolates was more robust than the PLY of D39 creating more damaging effects in a shorter time. Both toxins increased the resting membrane potential of recorded neurons; however, in the presence of the clinical isolate PLY, membrane potentials depolarized, raising them above the firing threshold for some neurons, while in the presence of PLY of D39, the effect was less pronounced (Figures 7C and 7D). Exposure of neurons to the toxins also affected neuronal firing properties. Action potential duration was significantly prolonged in the presence of either toxin (Figure S6A); however, action potential duration was more prolonged for neurons exposed to the PLY variant of the clinical isolates, almost doubling in duration (Figure S6B). Taken together, these data demonstrate that both PLY variants functionally impair striatal neurons in *ex vivo* mouse brain slices. However, neurons exposed to the PLY with the aa substitution are more robustly and quickly impaired than those exposed to the PLY of D39.

Based on all the *in vitro* and the *ex vivo* findings, we can conclude that the three pneumococcal meningitis clinical isolates express and release a PLY variant that differs only in one amino acid at position 380 within the D4 domain from the PLY of the reference strain D39, but also exhibits a significantly higher cytotoxicity toward neurons and, most likely, toward many other cells. Moreover, the evidence that the PLY variant of the meningitis clinical isolates, when in contact with neurons, induces the formation of more and larger pores than the original PLY variant of the reference strain D39, strongly suggests that a subtle point mutation-induced conformational change occurring in the D4 domain (Figure 4C) enhances the capability of D4 to anchor to the eukaryotic plasma membrane, thereby enhancing PLY’s pore-forming activity.

## Discussion

*S. pneumoniae* is the leading cause of bacterial meningitis, and the major burden of this disease is that up to 50% of the survivors suffer from permanent neurological disabilities due to neuronal damage caused by the infection.^11^^,^^12^^,^^13^^,^^14^ PLY is certainly a major pneumococcal virulence factor responsible for inflicting damage to neuronal cells.^4^^,^^5^^,^^6^ Inside neurons, PLY can alter mitochondrial membrane potential causing the release of apoptosis-inducing factor and ultimately leading to neuronal cell death.^5^ Moreover, PLY can also bind to exposed β-actin filaments on neuronal plasma membranes and, through this interaction, trigger β-actin polymerization and the formation of β-actin aggregates. This cascade can then result in cytoskeleton instability and, consequently, cell death because of cytoskeleton disruption.^6^ All these detrimental processes for neuronal cells that PLY is able to exert intracellularly are dependent on the capacity of either PLY or the bacteria to enter neuronal cells.^6^ As a toxin, PLY’s main feature is pore-forming activity, which is further supported from our data in this study where we demonstrate through electron microscopy PLY induced pore formation on the neuronal plasma membrane. Consequently, these breakings within the membrane enable pneumococci to invade neuronal cells.^6^ One of the main dogmas in pneumococcal cell biology has been that PLY is regarded as one of the most highly conserved proteins among all *S. pneumoniae* strains. Here we show an exception to this rule by proving that only a single amino acid change is enough to generate different variants of the same toxin with, consequently, different cytotoxic power. Taken together, our work here could explain why certain pneumococcal strains induce an elevated severity of neuronal damage during meningitis infection and neurological sequelae post-meningitis, while others do not.

The D4 domain is fundamental for the anchoring of PLY to the plasma membrane of the cell that PLY contacts. Recently, it was reported that the D4 domain binds to the membrane with our without cholesterol, a new and important concept in the field of pneumococcal biology;^7^ cholesterol makes the binding with the membrane irreversible, once D4 anchors to the membrane, D1-D3 PLY domains form the so-called pre-pore complex progressively leading to a vertical collapse of the pre-pore leads to the expansion of the ring diameter until the formation of the pore.^7^ The pore formation process is therefore initiated once D4 binds to the membrane, without which the pore is not formed. Here, we showed that the PLY variant expressed by the three pneumococcal meningitis clinical isolates had a single aa substitution in the D4 domain compared to the original WP_001284359.1-PLY deposited on the NCBI protein database. Knowing this, we can therefore speculate that the different cytotoxic power and different degrees of pore formation, both in terms of numbers and size of the pores, are likely due to the different binding strength of the D4 domain of the clinical isolates’ PLY variant to neuronal plasma membrane. Indeed, when examining the hydrogen bonding lost from the single Asp380-Asn420 mutation, and the subsequent introduction of a new steric clash when Asp380 is replaced by an asparagine residue, it is not hard to imagine this resulting in a structural rearrangement in this D4 region of the protein, which could ultimately (adversely) affect Domains 1–3 and PLY function/interactions with the neuronal membrane.

PLY belongs to the class of cholesterol-dependent cytolysins (CDCs); streptolysin O (SLO) is the CDC expressed by *Streptococcus pyogenes* (Group A Streptococci), another member of the *Streptococcus* genus. It was previously reported that different strains of Group A streptococci form different variants of SLOs.^20^ Notably, similar to what we reported for PLY in this study, targeted aa substitutions could alter the cytotoxicity of SLO.^21^ The Gram-positive *Listeria monocytogenes* is another etiological cause of bacterial meningitis.^22^ Listeriolysin (LLO) is the CDC expressed by *L. monocytogenes*, and it was previously reported that the phosphorylation of the PEST-like LLO sequence led to point mutations in that region resulting in increased cytotoxicity or attenuated virulence.^23^ Considering our new findings and the current state-of-the-art literature on CDCs for other streptococcal and meningeal pathogens, it is therefore correct to conclude that, even though CDCs such as PLY, SLO and LLO are highly conserved, single aa differences can have a great impact on the cytotoxic power of CDCs.

Because of the acknowledged concept of the high conservation of CDCs among bacterial strains, CDCs were reported to elicit an important neuroinflammatory response upon brain infection, thus CDCs were proposed as candidates for adjuvant therapy to fight bacterial meningitis.^24^ Detoxified versions of PLY have been considered good vaccine candidates against invasive pneumococcal disease.^25^ Protection against pneumococcal pneumonia was also achieved by immunizing mice with antibodies against PLY.^26^ Yet, our new findings showed that one single aa change can greatly alter virulence and toxicity, therefore, our study sheds light to the concept that one PLY version may not be enough to generate protection against several pneumococcal serotypes.

### Limitations of the study

The main limitation of the study lies in the number of the *S. pneumoniae* clinical isolates investigated. The focus on specific serotypes (6A, 15A, and 16F) may give only a limited overview of the PLY expressed by all clinical strains that cause pneumococcal meningitis globally. We have the availability of more clinical isolates from patients with meningitis; unfortunately, some of them were not able to grow in the liquid medium, therefore we could not use those for the experiments performed in this study.

## STAR★Methods

### Key resources table

**Table:** 

REAGENT or RESOURCE	SOURCE	IDENTIFIER
**Chemicals, peptides, and recombinant proteins**		

EMEM L-glutamine	ATCC	D1603
F-12 Nut Mix	Gibco	21765-029
TrypLE Express	Gibco	12605-010
PBS ph 7,4	Gibco	10010023
Trypan Blue Staining 0,4%	Gibco	15250061
CyQUANT LDH Cytotoxicity Assay	Invitrogen	C20301
Retinoic Acid	Biotechne	0695
FBS, Qualified	Gibco	10270-106
Pen Strep	Gibco	15140-122
Sodium phosphate dibasic	Sigma	7558-79-4
Sodium chloride solution	Sigma-Aldrich	16818
Imidazole	Sigma-Aldrich	I2399
HisPur Ni-NTA Magnetic	Thermo Scientific	88831
Cholesterol, Highly purified	EMD Millipore	228111-5GM
Absolut Ethanol	Histolab	01399.01L
Ethanol	Emsure	I834609623
Triton X	Sigma-aldrich	9036-19-5
Acedic acid glacial 100%	Merck	K23348663639
NuPage antioxidant	Invitrogen	NP0005
MagicMark	Invitrogen	LC5602
PageRuler Plus Prestained Protein Ladder	Thermo Scientific	2709121
NuPage LDS Sample Buffer (4X)	Invitrogen	NP0007
InstantBlue Coomassie Protein Stain	Abcam	ISB1L
Tween 20	Sigma	P1379-250ML
NuPAGE 4-12% Bis-Tris Gel	Invitrogen	NP0322BOX
DEAE-Sepharose	Sigma-aldrich	DCL6B100
Todd-Hewitt Broth w/ 2% Yeast Extract (THY)	Karolinska University Hospital	MIK4004- 1000
Glycerol 50%	Karolinska University Hospital	MIK1941
Isofurane, Forene	AbbVieAB (Apoteket)	506949
Freund incomplete adjuvant	Sigma-aldrich	F5506
Protein A-Sepharose	Sigma-aldrich	101090
LytA	Sven Hammerschmidt’s group Greifswald University, Germany	Not commercially available
Recombinant PLYs	Protein Production Platform, Nanyang Technological University, Singapore	Not commercially available

**Antibodies**		

Anti-LytA antibody	Sven Hammerschmidt’s group Greifswald University, Germany	Not commercially available
Anti-PLY antibody	Abcam	Ab71811
Alexa Fluor Goat anti Mouse 488 secondary antibody (for the detection of PLY in immunofluorescence microscopy)	Thermo Fisher Scientific	A-11001
HRP-goat anti mouse secondary antibody (for the detection of PLY and LytA in western blot)	Invitrogen/Thermo Fisher Scientific	31430

**Biological samples**		

Articifial CSF (ACSF)	Tocris	3525

**Bacterial and virus strains**		

*S. pneumoniae* D39	Sven Hammerschmidt’s group Greifswald University, Germany	Not available (*S. pneumoniae* reference strain for PLY)
*S. pneumoniae* meningitis clinical isolates serotype 6A/15A/16F	Federico Iovino’s group, Karolinska Institutet, Sweden	Not available (*S. pneumoniae* strains from reference nr 9, originally obtained from Prof. Diederik van de Beek’s group at the Academic Medical Center, Amsterdam, The Netherlands)

**Experimental models: Cell lines**		

SH-SY5Y neuroblastoma cell line	ATCC	9036-19-5

**Deposited data**		

PLY aa sequence *S. pneumoniae D39*	NCBI Protein	ABJ53672
PLY aa sequence meningitis clinical isolates and TIGR4	NCBI Protein	AAK75991

**Software and algorithms**		

Prism	GraphPad Software	Karolinska Institutet IT

### Resource availability

#### Lead contact

Further information and requests for resources should be directed to and will be fulfilled by the lead contact, Federico Iovino (federico.iovino@ki.se).

#### Materials availability

This study generated the pneumolysin nucleotide and amino acid sequences of the meningitis clinical isolates used, the sequence results are available in the results and supplemental information. The PLY sequence with the aa replacement is deposited on the NCBI Protein database accession number AAK75991.

#### Data and code availability

All raw data are available on Mendeley Data (https://data.mendeley.com/preview/9py9n93rs2) and are available upon request to the lead contact.

### Experimental model and study participant details

The three pneumococcal clinical isolates used in this study were originally isolated at the Amsterdam Academic Medical Center (group of Prof. Diederik van de Beek) from patients that died of pneumococcal meningitis. The biological replicates of the experiments performed with SH-SY5Y-derived neuronal cells were performed with cells at a different culture passage for each replicate. The biological replicates of the western blots were performed with different lysates for each replicate. For the patch clamp electrophysiological recordings, each brain slice was obtained from a different mouse. Animal experiments performed at Karolinska Institutet were performed according to the ethical permit nr 2022-2020 approved by the local ethical committee *Stockholms Norra djurförsöksetiska nämnd*; animal experiments performed at the University of Greifswald were performed according to the LALLF M-V permit nr. 7221.3-2-008/23-2 approved by *Landesamt für Landwirtschaft, Lebensmittelsicherheit und Fischerei Mecklenburg–Vorpommern* (LALLFV M-V, Rostock, Germany) and the LALLFV M-V ethical board. All figure legends report the exact n power of each experiment performed. All the key resources described below in the methods are summarized in key resources table.

### Method details

#### Cultivation of Streptococcus pneumoniae and growth curves in THY and human blood

*Streptococcus pneumoniae* D39 (serotype 2) laboratory strain, the isogenic mutant D39Δ*ply*, and the pneumococcal meningitis clinical isolates serotyped 6A, 15A and 16F^8^ were grown at 37°C with 5% CO_2_ to an OD_620_ of 0.3-0.35 (exponential phase) in Todd-Hewitt broth (THY) and stored in a solution of THY supplemented with 20% glycerol at -80°C. The pneumococcal strain D39Δ*ply* was constructed by insertion-duplication mutagenesis as previously described.^27^ For growth experiments of pneumococcal liquid cultures in THY, for all pneumococcal strains bacterial suspensions were made in 1 mL volume with 900 μL THY and 100 μL liquid culture taken from a pre-grown aliquot, aliquot made as described above; each pneumococcal strain was grown in triplicate (three separate 1 mL-cultures), incubated at 37°C with 5% CO_2_ and growth was measured 620 nm with Novaspec III+ spectrophotometer (Thermo Fisher Scientific) every 30 minutes. For growth experiments of pneumococcal liquid cultures in human blood, for each pneumococcal strain 450 μL of blood were transferred into a 1.5 mL-Eppendorf tube and tubes were then placed in a rotator for 40 rpm-constant vertical rotation at 37°C with 5% CO_2_ to prevent clotting; for each pneumococcal strain, 50 μL of liquid culture was taken from a pre-grown aliquot in THY, centrifuged at 10,000 rpm for 3 minutes, THY supernatant discarded and bacterial pellet was resuspended in blood; 450 μL of blood were added to each 50 μL of bacterial suspension (in blood) and incubated at 37°C with 5% CO_2_; bacterial growth was assessed by count of colony forming units (CFU) after 2, 4, 6 and 8 hours growth.

#### Culture of neuronal-like cells and *in vitro* cytotoxicity assays

Human SH-SY5Y neuroblastoma cells were cultured as previously described.^6^ Briefly, cells were seeded in 12 well plates at a density of 200.000 cells/mL, differentiated for 12 days using differentiation media made of EMEM (ATCC) and F12 (Gibco) in 1:1 ratio, 1% fetal bovine serum (FBS) (Gibco), 1% penicillin/streptomycin (Gibco), and 10μM retinoic acid (RA) (Biotchne) and kept in incubator at 37°C with 5% CO_2_. The day before toxin inoculation, neuronal-like cells were put in differentiation media without antibiotics (EMEM and F12 in 1:1 ratio, 1% FBS, 10μM RA). The day of the experiment, the medium was discarded, and cells were put in EMEM and F12 in 1:1 ratio, 1% FBS, incubated 1h at 37°C, 5% CO_2_. For the infection experiments in Figure S3, THY-grown 1 mL aliquots of pneumococcal strains (OD_620_ of 0.3-0.35) were used; neuronal-like cells were then infected with Multiplicity of Infection (MOI)=20 for D39 and D39Δ*ply*, while the MOI of the serotypes 6A, 15A and 16F was adjusted multiplying the MOI of 20 by the coefficient factors in Table S1 in order to match the same amount of released PLY for all strains; coefficient factors were obtained first calculating the ratio of PLY in the supernatant (PLY in the supernatant of D39 was set as 1) using the average values of the GAPDH/PLY ratio values of the clinical isolates and the laboratory strain D39; for each clinical isolate, this number was used to calculate the coefficient factor to match the PLY in the supernatant of D39 (Table S1). For the cytotoxicity assays with recombinant PLY, neuronal-like cells were incubated with 1 μg/mL of either PLY of the laboratory strain D39 or PLY from meningitis clinical serotypes, while cells treated with lysate buffer (Invitrogen) or untreated were the positive and negative controls respectively. Three timepoints were tested: 1 hour, 2 hours and 4 hours incubation; for each timepoint, 50 μl of each sample (=well) were transferred to a 96-well flat-bottom plate, in technical triplicates. Three biological samples were analyzed. During the assay, supernatant aliquots were kept at 4°C, neuronal-like cells were kept at 37°C with 5% CO_2_. CyQUANT LDH Cytotoxicity Assay Kit (Invitrogen, Cat. C20300 and C20301) was used to measure the released lactase dehydrogenase (LDH), as assessment of cytotoxicity. Absorbance was measured with Multiskan FC microplate reader (Thermo Fisher Scientific) at 450 nm and 620 nm; to determine LDH activity, 620 nm absorbance value (background from the instrument) was subtracted to 450 nm absorbance value.

#### Expression and purification of recombinant PLY

Large scale cultivation of *Escherichia coli* BL21 (DE3) containing the pRARE plasmid was performed in a LEX system (Harbinger Biotech). Using glycerol stocks, inoculation cultures were started in 20mL TB media supplemented with appropriate antibiotics. The cultures were incubated at 37°C, 200 rpm overnight. The following morning, bottles of 750 mL Terrific Broth (TB) supplemented with appropriate antibiotics and 100 μL of antifoam 204 (Sigma-Aldrich) were inoculated with the inoculation cultures. The cultures were incubated at 37°C in the LEX system with aeration and agitation through the bubbling of filtered air through the cultures. When the OD_600_ reached ∼2, the temperature was reduced to 18°C and the cultures were induced after 30 to 60 minutes with 0.5 mM IPTG. Protein expression was allowed to continue overnight; cells were harvested by centrifugation at 4200 rpm at 15°C for 10 minutes. The supernatants were discarded, and the cells were resuspended in lysis buffer, 1.5 times of the cell pellet weight, at 200 rpm, 4°C for approximately 30 minutes (Lysis buffer composition: 100 mM HEPES, 500 mM NaCl, 10 mM Imidazole, 10 % glycerol, 0.5 mM TCEP, pH 8.0 Benzonase – 4 μL per 750 mL cultivation - 250U/μL from Merck, Protease Inhibitor Cocktail Set III EDTA-free - 1000x dilution in lysis buffer - from Calbiochem). The cell suspensions were stored at -80°C. The re-suspended cell pellet suspensions were thawed and sonicated (Sonics Vibra-cell) at 70% amplitude, 3 seconds on/off for 3 minutes on ice. The lysate was clarified by centrifugation at 47000 g, 4°C for 25 minutes. The supernatants were filtered through 1.2 μm syringe filters and loaded onto AKTA Xpress system (GE Healthcare). The purification regime is briefly as follows. The lysates were loaded on IMAC columns. The columns were washed with 20 CV of wash 1 and 20 CV of wash 2 buffer or until a stable baseline was obtained respectively. The eluted proteins were collected and stored in sample loops on the system and then injected into Gel Filtration (GF) columns. Elution peaks were collected in 2 mL fractions and analyzed on SDS-PAGE gels. The entire purification was performed at 4°C. All IMAC columns are stripped of Ni^2+^, cleaned-in-place with 0.5 M NaOH, re-charged with Ni^2+^ (0.1 M NiCl_2_) and stored in 20% ethanol in between purification runs. All GF columns are cleaned-in-place with 0.5 M NaOH and stored in 20% ethanol in between purification runs. Relevant peaks were pooled, TCEP was added to a total concentration of 2 mM. The protein sample was concentrated in Vivaspin 20 filter concentrators (VivaScience) at 15°C to approximately 15 mg/mL. (< 18kDa – 5K MWCO, 19-49 kDa – 10K MWCO, >50kDa – 30K MWCO). The final protein concentration was assessed by measuring absorbance at 280nm on Nanodrop ND-1000 (Nano-Drop Technologies). The final protein purity was assessed on SDS-PAGE gel. The final protein batch was then aliquoted into smaller fractions, frozen in liquid nitrogen and stored at -80°C.

#### Expression and purification of LytA, and generation of antibodies against LytA

The pneumococcal gene encoding LytA (spd_1737, from strain *S. pneumoniae* D39) was amplified by PCR using primer lytAforNheI 5′ GCGCGCTAGCGAAATTAATGTGAGTAAATTAAGAACAG 3′ and lytArev2HindIII 5′ GCGCAAGCTTATTTTACTGTAATCAAGCCATCTGGC 3′. After digestion of the PCR and the target vector pTP1 with NheI and HindIII, gene was ligated into the IPTG-inducible expression vector pTP1.^28^ For protein expression, *E. coli* BL21 DE3 was transformed with the resulting plasmid. Heterologously expressed LytA was purified using DEAE sepharose as described.^29^

Polyclonal antibodies against pneumococcal LytA were raised in mice using routine immunization protocols. Briefly, 20μg of heterologously expressed protein in 100 μL PBS and Freund incomplete adjuvant (50:50 v/v) (Sigma-Aldrich) were injected intraperitoneally. Female CD-1 mice (Janvier) were boosted twice (at days 14 and 28) with 20 μg of protein and Freund incomplete adjuvant (50:50 v/v) (SigmaAldrich). After bleeding, the polyclonal IgGs from serum were purified using protein A-Sepharose (Sigma-Aldrich). Animal experiments were approved by the *Landesamt für Landwirtschaft, Lebensmittelsicherheit und Fischerei Mecklenburg–Vorpommern* (LALLFV M-V, Rostock, Germany) and the LALLFV M-V ethical board (LALLF M-V permit nr. 7221.3-2-008/23-2).

#### Western blot analysis and coomassie staining

200 μl of THY-grown pneumococcal strains (OD_620_ of 0.4) were centrifuged at 10,000 rpm for 5 minutes, bacterial pellet was then separated from the supernatant; both pellet and supernatant were resuspended with LDS sample buffer (Thermo Fisher Scientific) to reach 1X concentration and boiled at 95°C for 10 minutes; pellet and supernatant samples were then loaded into NuPage Novex 4-12% Bis-Tris SDS-PAGE Gels (Thermo Fisher Scientific), electroblotting was performed using the Mini Gel Tank (Thermo Fisher Scientific). PVDF membranes were first incubated with PBS-T (T=0,1% Tween) supplemented with 5% milk for one hour at room temperature (RT); after washing with PBS-T, membranes were incubated with primary antibodies (1:1000) was performed overnight, incubation with secondary antibodies (1:5000) was performed for one hour. Both primary and secondary antibodies were diluted in PBS-T supplemented with 1% dry milk. Protein bands were detected by incubating membranes with ECL Prime Western Blotting Detection Reagents (Cytiva, Thermo Fisher Scientific) and imaged using ImageQuant LAS 4000 (GE Healthcare). For Coomassie stainings, after electrophoresis gels were incubated with InstantBlue Coomassie Protein Stain (Abcam) for 45 minutes and then photographed. The intensity of protein bands on PVDF membranes and Coomassie-stained-gels was measured by Image J as previously described.^8^ PLY expression was calculated as a ratio of the PLY band intensity divided by the GAPDH band intensity; band intensities were measured by Image J.

#### Pull-down experiments with recombinant PLY and purified cholesterol

To perform the pull-down experiment using magnetic His-Pur Ni-NTA magnetic beads (Thermo Fisher Scientific), the pH of the Equilibration Buffer, Wash Buffer, and Elution Buffer (see reagents in the key resources table) were initially adjusted to 8.0. The beads were mixed to ensure homogeneity of the beads in solution. Maintaining everything on ice, 160 μL of Equilibration Buffer was added to 40 μL of beads, vortexed and placed onto a magnetic rack. Beads were collected against the side of the tube and after 1 minute the supernatant was discarded. Recombinant His-tag-PLYs, either of D39 strain or clinical isolates, was bound to magnetic beads in 500 μL lysis buffer for 45 minutes, on orbital shaker at 10 rpm at 4°C. The control group was prepared without the addition of any PLY in this step (beads only). The washing step was performed using 500 μL washing buffer; 400 μL of Equilibration Buffer was add to the tube, and vortexed for 10 seconds. The beads were collected by placing the Eppendorf tubes onto a magnetic rack; the supernatant was discarded. The pull-down sample was prepared by diluting the cholesterol solution with an equal volume of Equilibration Buffer 1:1. 300 μg of purified cholesterol were diluted in 500 μl warm ethanol; 400 μL of prepared 1:1 PLY-cholesterol solution was added to the beads tube. The beads were vortexed for 10 seconds, then incubated onto a vertical rotator (10 rpm rotation) for 2 hours at 4°C. The beads were then collected by placing the Eppendorf tubes onto a magnetic rack. Two washing steps were performed by adding 400 μL of Wash Buffer to the tubes, then vortexed for 10 seconds. The beads were collected onto the magnetic rack, while the supernatant was removed and discarded. The elution was carried out by adding 25 μL of Elution Buffer to the tube and vortexed for 15 seconds. Beads were incubated for 15 minutes on a vertical rotor at 4°C. The beads were collected onto a magnetic stand. The supernatant was removed and saved for the cholesterol concentration analysis. Amounts of cholesterol bound by PLY were measured using the Cholesterol Quantification Assay Kit (Merck).

#### Immunofluorescence stainings and microscopy analysis

For all pneumococcal strains, growth in blood was first performed in 1 mL-volume as described above (section “Cultivation of Streptococcus pneumoniae and growth curves in THY and human blood”) and stopped after 8 hours; 1 mL-cultures (in 1.5 mL-Eppendorf tubes) were centrifuged at 10,000 rpm for 5 minutes to harvest the serum containing the released PLY. For PLY staining of serum samples, drops of 5 μl serum were pipetted onto microscope glass slides and let dry at room temperature; the edges around each drop were marked with PAP pen and incubated with mouse anti PLY antibody (Abcam) for 1 hour at RT, followed by Alexa Fluor 594 goat anti-mouse (Thermo Fisher Scientific) for 1 hour at RT in the dark. Stained drops were imaged using a Zeiss Observer.Z1 fluorescence microscope with Orca-Flash4.OLT camera; 5 μl drops were imaged with 5X objective combined with the function "Tiles" of the imaging program Zen 2 (Zeiss). PLY fluorescence signal was quantified using the software Image J, as previously described.^8^^,^^30^ Briefly, firstly all fluorescence images were converted into grayscale images; with the function "Threshold," the area covered by the fluorescence signal was defined with the function "Create Selection" and then quantified with the "Measure" function.

#### PCR reactions

Pneumococcal strains (D39 laboratory strain and clinical isolates) were grown on blood agar plates at 37°C and 5% CO_2_ for 8-10 hours. A sterile swab was used to transfer bacterial material into tubes containing 100μl phosphate buffered saline (PBS, pH 7.4). Bacteria were heated for 10 minutes at 98°C and afterwards centrifuged for 1 minute at 13.000 x g. 10 μL of the supernatant (containing chromosomal DNA) was used as template for the PCR reactions. To amplify the *ply* gene region (*ply* + approx. 250 bp up- and downstream) primer *ply*_check_f (5′ GAACTTTATGATAGAAGAGCCGG 3′) and primer *ply*_check_r (5′ GATATAAACAGCAAAATATTTCCG 3′) were used. Finally, PCR products were purified using a PCR-clean up kit (Promega).

#### Gene sequencing, amino acid sequence analysis and structural modeling

The Sanger sequencing was performed on an ABI 3730 sequencer (Applied Biosystems) using the BigDye Terminator v3.1 Cycle Sequencing Kit (Applied Biosystems) according to manufacturer’s instructions. PCR sequences were analyzed and visualized using the Multalign online tool.^31^ For the conversion of gene to amino acid sequences, *ply* gene sequences were first aligned using Clustal Omega^32^ and uploaded into ESPrit 3.0^33^ for display output. Structural modeling was done using the deposited PLY structure (PDB code 5AOD; deposited by van Pee, K. and Yildiz, O.) with the program PyMol (Schrödiger, Inc., CA).

#### Scanning electron microscopy (SEM) and measurement of pore sizes

SH-SY5Y-differentiated neuronal cells were treated with 1 μg/mL of either PLY of the laboratory strain D39 or PLY from the meningitis clinical isolates for 30 minutes at 37°C with 5% CO_2_, untreated cells were used as negative control. Cells grown on glass round coverslips (Fisher Scientific) were fixed by immersion in 2.5 % glutaraldehyde (Ladd Research) in 0.1M phosphate buffer, pH 7.4. The coverslips were briefly rinsed in phosphate buffer pH 7.4 followed by MilliQ water prior to stepwise ethanol dehydration and critical-point-drying using carbon dioxide (Leica EM CPD 030). The cover slips were mounted on alumina SEM pins using carbon conductive tabs (Ted Pella) and sputter coated with a 10 nm layer of platina (Quorum Q150T ES). SEM images were acquired using an Ultra 55 field emission scanning electron microscope (Zeiss) at 3 kV and the SE2 detector. Manual identification of plasma membrane pore was performed using the “straight, segmented lines” function of Image J. To ensure accuracy, a subset of images was randomly selected for validation. The pixel values corresponding to the length of each straight line were converted to micrometers using the known scale of the images.

#### Preparation of mouse brain slices and patch clamp electrophysiological recordings

SERT-cre wt/wt male 8-week-old mice were anesthetized with isoflurane (VM Pharma AB, Sweden). Once mice were confirmed to have lost consciousness and sensation to pain, they were decapitated, and the brain was removed. Animal experiments were performed according to the ethical permit nr 2022-2020 approved by the local ethical committee *Stockholms Norra djurförsöksetiska nämnd*. Brains were then transferred to ice cold cutting solution and parasagittal slices, 250μm in thickness, were cut using a VT1200S Vibratome (Leica, Japan). The cutting solution contained (in mM): 205 sucrose, 10 glucose, 25 NaHCO_3_, 2.5 KCl, 1.25 NaH_2_PO_4_, 0.5 CaCl_2_ and 7.5 MgCl_2_ and was saturated with 95% oxygen and 5% carbon dioxide. Following cutting, slices were transferred to a chamber where they were incubated for 30 minutes in artificial cerebrospinal fluid (ACSF, Biotechne-Tocris) at 35°C. The ACSF was saturated with 95% oxygen and 5% carbon dioxide and contained (in mM): 125 NaCl, 25 glucose, 25 NaHCO_3_, 2.5 KCl, 2 CaCl_2_, 1.25 NaH_2_PO_4_, 1 MgCl_2_. Prior to electrophysiological recordings taking place, slices were kept at room temperature for at least 1 hour. Electrophysiological recordings were obtained from striatal neuron somas in oxygenated ACSF at 34–35°C. Neuron somas were visualized using infrared differential interference contrast (IR-DIC) microscopy on a BX51WI (Olympus, Japan) upright microscope using a 40X long-working-distance immersion objective and a digital camera (Hamamatsu Photonics, Japan). Whole-cell patch clamp and cell-attached recordings were obtained using borosilicate pipettes with resistances of 6–10 MOhm, pulled with a Flaming / Brown micropipette puller P-1000 (Sutter Instruments Co, Novato, CA). Pipettes were filled with an intracellular solution containing (in mM): 130 K-gluconate, 5 KCl, 10 Na2-Phosphocreatine, 10 HEPES, 4 ATP-Mg, 0.3 GTP-Na and with pH adjusted with KOH to 7.2 and osmolarity of 280–290 mOsm/L. Throughout these recordings, pipette capacitance and access resistance were compensated for and the quality of the seal was monitored throughout to make sure the pipette did not lose contact with the cell. Recordings were performed using Multiclamp 700B amplifiers (Molecular Devices, CA, USA), filtered at 2 kHz, digitized (10–20 kHz) using ITC-18 (HEKA Elektronik, Instrutech, NY, USA), and acquired and analysed using custom-made programs in Igor Pro (Wavemetrics, OR, USA). To assess the effect of the two PLY variants on intrinsic neuronal properties, 250 μl of either PLY concentrated 10 μg/mL was added to a beaker of 100 mL ACSF and left to wash in until neuronal health began to deteriorate. Prior to addition of PLY, baseline intrinsic neuronal properties were obtained to assess both the initial health of the neuron and impact of the toxin on neuronal health. Intrinsic neuronal properties were extracted by recording neuronal responses to a series of standardised hyperpolarizing and depolarizing current injections in whole cell current clamp mode with baseline membrane potentials held at -75 mV.

### Quantification and statistical analysis

Analysis was performed using Prism GraphPad software. For all multiple comparisons (more than two groups), 2-tails ANOVA test was run to assess the presence of differences between the groups, and then a Dunn's test was applied for pairwise comparisons; unpaired t-tests were performed for pairwise comparisons with normal distribution, Wilcoxon tests were performed for pairwise comparisons without normal distribution (pairwise comparisons = experiments performed with only two experimental groups). Differences were defined as “significant” and were assigned one asterisk (∗) when p < 0.05, two asterisks (∗∗) when p < 0.01, three asterisks (∗∗∗) when p < 0.001, four asterisks (∗∗∗∗) when p < 0.0001; exact p values (with three decimal numbers) were generated by Prism Graph Pad software when p values were < 0.05 or 0.01 and are reported in the figure legends. F, T, R^2^ values were generated by Prism Graph Pad software in case of group comparisons and t-tests with normal distribution.

## Supplemental information

Document S1. Figures S1‒S6 and Table S1
