# Supplementary material for: The single D380 amino acid substitution increases pneumolysin cytotoxicity toward neuronal cells
Source: iScience. 2024 Mar 27;27(4):109583. doi: 10.1016/j.isci.2024.109583 (PMC11022043; doi:10.1016/j.isci.2024.109583)
Supplement: Document S1. Figures S1‒S6 and Table S1 [file mmc1.pdf]

iScience, Volume *27*

Supplemental information

The single D380 amino acid substitution increases pneumolysin cytotoxicity toward neuronal cells

Simona Serra, Vittorio Iannotti, Margherita Ferrante, Miguel Tofiño-Vian, Joseph Baxendale, Gilad Silberberg, Thomas P. Kohler, Sven Hammerschmidt, Andrew T. Ulijasz, and Federico Iovino

The single D380 amino acid substitution increases pneumolysin cytotoxicity towards neuronal cells

Simona Serra[1], Vittorio Iannotti[1], Margherita Ferrante[1], Miguel Tofiño-Vian[1], Joseph Baxendale[1], Gilad Silberberg[1], Thomas P. Kohler[2], Sven Hammerschmidt[2], Andrew T. Ulijasz[3], Federico Iovino[1]

[1] Department of Neuroscience, Karolinska Institutet, Stockholm, Sweden.

[2] Department of Molecular Genetics and Infection Biology, Interfaculty Institute for Genetics and Functional Genomics, Center for Functional Genomics of Microbes, University of Greifswald, Greifswald, Germany

[3] Department of Microbiology and Immunology, Loyola University Chicago, Maywood, IL USA

Correspondence: Federico Iovino, federico.iovino@ki.se

Supplemental Information

Supplemental Figure S1. Growth of pneumococci in THY. The three pneumococcal meningitis clinical isolates presented a growth curve in THY similar to the reference strain D39; growth experiments were performed three times; error bars represent standard deviations calculated using the three OD_{620} values at each time point. THY-grown pneumococci were then used for the quantification of released PLY shown in Figure 1.

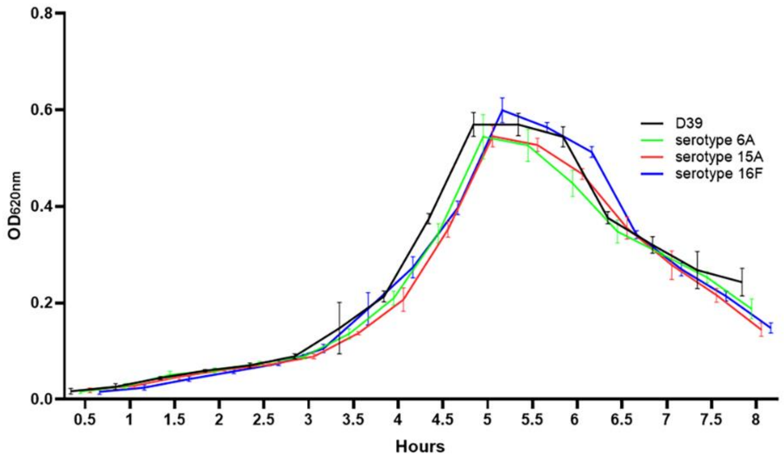

Supplemental Figure S2. Growth of pneumococci in human blood. Also in whole human blood, the pneumococcal meningitis clinical isolates showed a growth pattern similar to the reference strain D39; growth was assessed by CFU count at different time points of growth; growth experiments in human blood were performed three times, each bar represents the average value and error bars represent standard deviations calculated using three growth values (CFU/mL) at each time point for each pneumococcal isolate/strain (each growth value was an average value calculated using the growth value of three technical replicates per each isolate/strain). No statistical differences were observed between the pneumococcal isolates/strains at each time point of growth. This finding complements the findings shown in Supplemental Figure S1, once assessed similar growth patterns bacteria were then used to quantify the release of PLY, as shown in Figure 1.

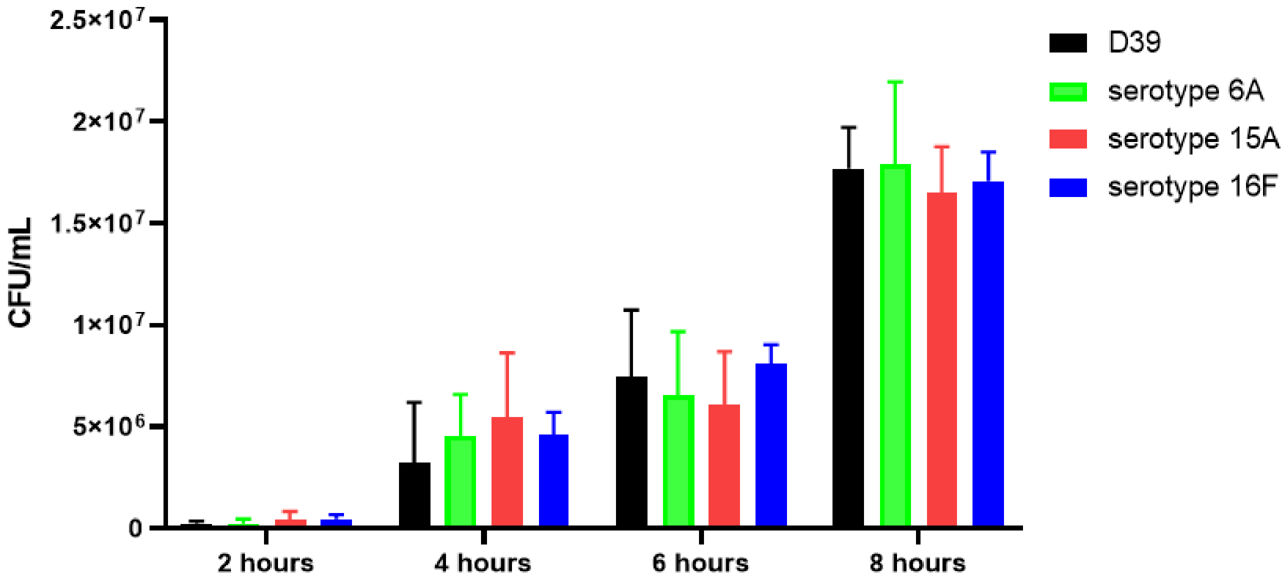

Supplementary Figure S3. Cytotoxicity of *S. pneumoniae* strains towards neuronal cells. LDH release assay using SH-SY5Y-derived neuronal cells infected with the reference strain D39, the isogenic mutant D39Δ*ply*, and the three pneumococcal meningitis clinical isolates serotypes 6A, 15A and 16F; infection doses (MOIs) were calculated in order to match the same amount of PLY in the supernatant (released) based on the western blot results shown in Figures 1A and B; three biological replicates (n = 3), with two technical replicates in each biological replicate, were performed, bars represent average values (average values for each group in each biological replicate were calculated using the cytotoxicity values of the two technical replicates for each isolate/strain), error bars represent standard deviations; * = $p<0.05$, ** = $p<0.01$ (2-tails ANOVA test was run to assess the presence of differences between the groups, and then a Dunn's test was applied for pairwise comparisons; p = 0.036 for comparison between D39 and serotype 6A, p = 0.022 for comparison between D39 and serotype 16F, p = 0.0052 for comparison between D39 and serotype 15A; Group comparison F = 7.433, T = 14.32; R^2 = 0.563, Degree of Freedom = 12). This pattern of cytotoxicity goes in line with the pattern of PLY release shown in Figure 1.

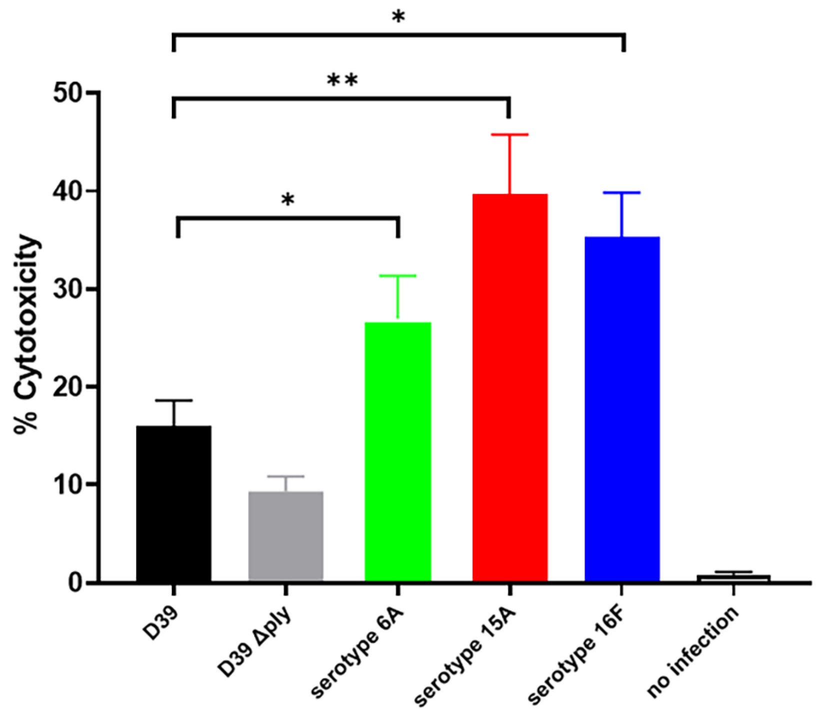

Supplementary Figure S4. ***S. pneumoniae*** **TIGR4 serotype 4 strain shares the same PLY variant of the pneumococcal meningitis clinical isolates.** Clustal Omega alignment of the PLY aa sequence of TIGR4, D39 and the three clinical isolates. The critical D380>N mutation is boxed in white. This PLY aa sequence alignment complements the same analysis performed comparing D39 with the meningitis clinical isolates shown in Figure 5.

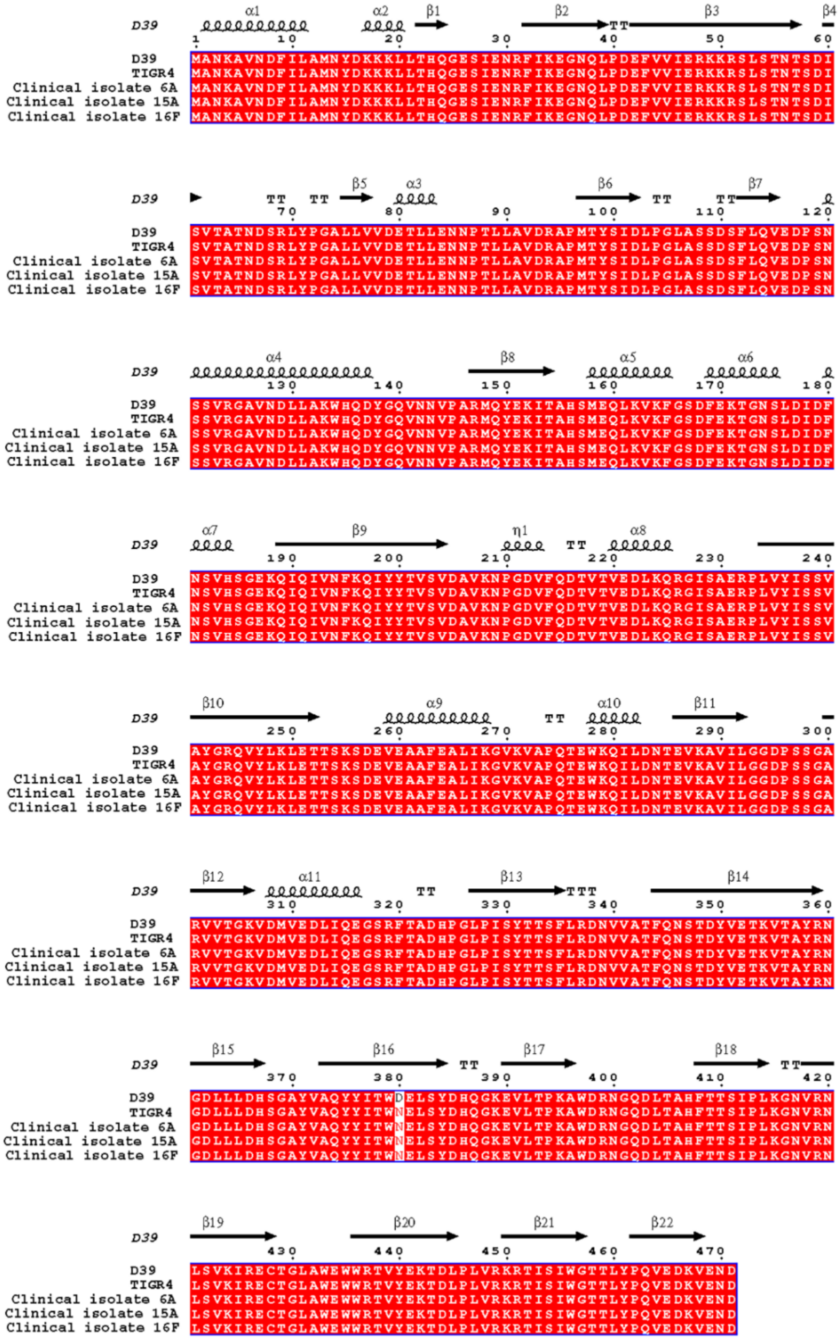

Supplementary Figure S5. Western blot-based quantification of PLY coupled to Ni-NTA beads. (**A**) PLY was detected at approximately 53-55 kDa by western blot analysis in PLY elution fraction from the beads after the pull-down reactions with purified cholesterol. (**B**) Quantification of PLY bound to the beads; PLY band intensities were measured by Image J; each datapoint represents the PLY band intensity of one western blot (= one biological replicate, n = 3), columns represent average values, error bars the standard deviations; ns = not-significant ($p > 0.05$). (**C**) Quantification of the "PLY of clinical isolate/D39 PLY" ratio calculated for the three biological replicates (n = 3) of the pull-down experiment (each datapoint represents one biological replicate, columns are average values, error bars the standard deviations); in order to match the bound cholesterol to the same quantities of PLY bound to the beads, the amount of cholesterol bound by D39 PLY showed in Figure 6D was adjusted according to the ratio values of D39 PLY in Supplementary Figure 5C; ratio values of PLY of clinical isolates were set to 1. Data shown in Supplementary Figure S5 complement the results shown in Figure 6D.

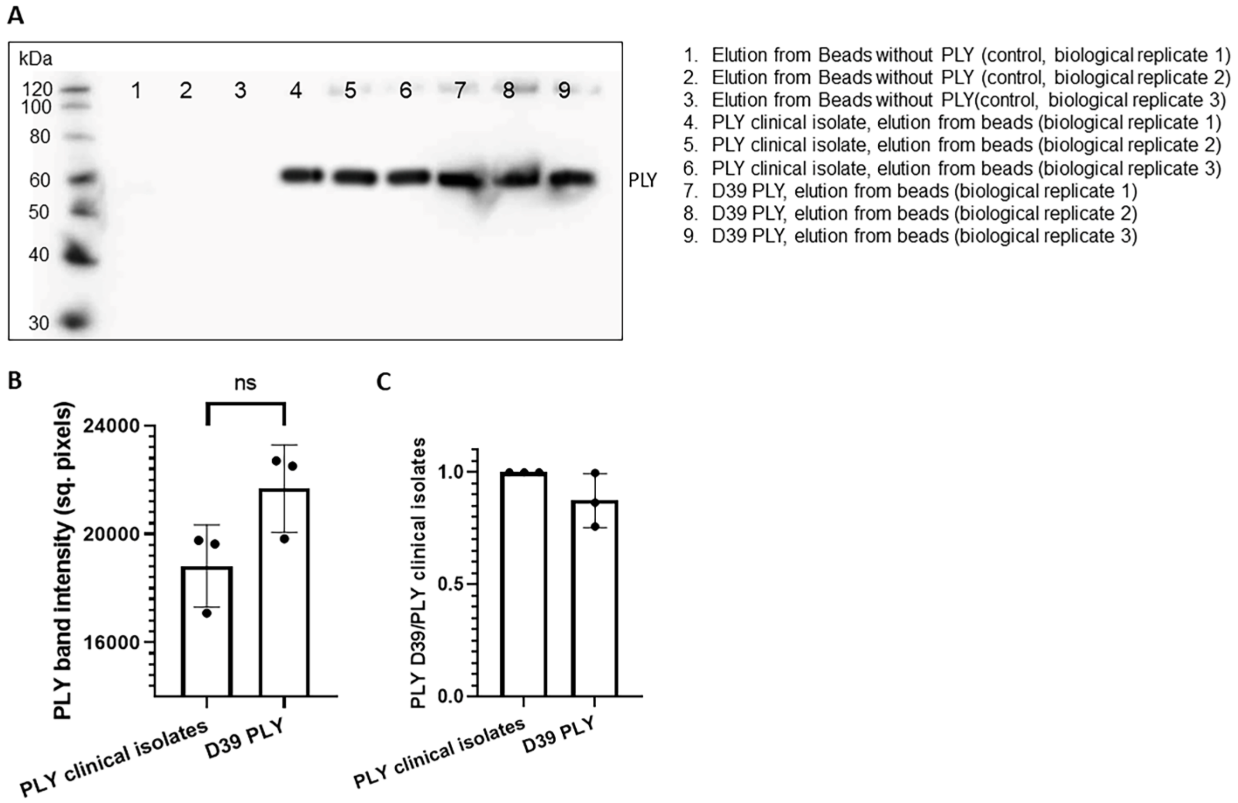

Supplementary Figure S6. Higher cytotoxicity of the PLY of meningitis clinical isolates towards striatal neurons assessed by *ex vivo* whole cell patch clamp electrophysiological recordings. Exposure of neurons to PLY affected neuronal firing properties. (**A**) Action potential duration was significantly prolonged in the presence of either PLYs (n = 8 nr of neurons in brain slices incubated with ACSF following bath-application of PLY of clinical isolates, n = 5 nr of neurons in brain slices incubated with ACSF following bath-application of PLY of D39); * = p< 0.05 (Data were in normal distribution and unpaired t-test was applied, p = 0.023 for PLY of clinical isolates and p = 0.031 for D39 PLY; F = 1.072, T = 2.273, R^2 = 0.291; Degree of Freedom = 15). (**B**) Normalized changes in action potential durations as shown in Supplementary Figure 6A; Notably, AP duration has doubled following exposure to the PLY variant of the meningitis clinical isolates. Data shown in Supplementary Figure S6 are additional data of the patch clamp electrophysiological recordings on mouse brain slices shown in Figure 7.

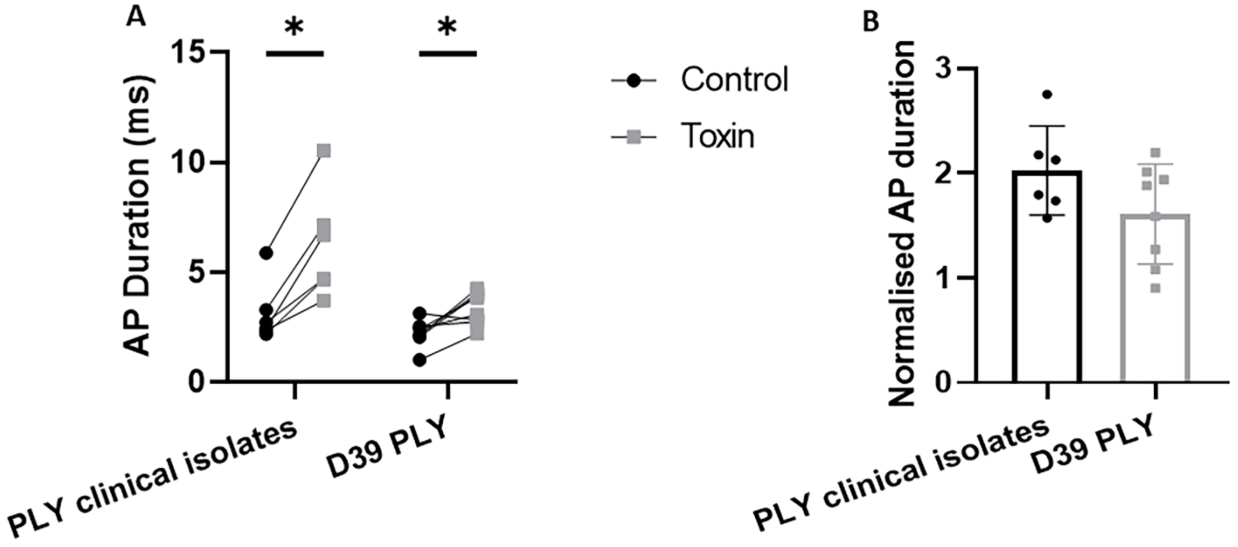

Supplemental Table S1. The percentage difference of the released PLY in the pneumococcal meningitis clinical isolates compared to the reference strain D39. The average value of the GAPDH/PLY ratio of the clinical isolates and the reference strain D39 from the western blot analysis in Figure 1B were used to calculate the ratio of PLY in the supernatant of each clinical isolate based on the PLY in the supernatant of D39; for each clinical isolate, the coefficient factor to match the released PLY (in the supernatant) of D39 was calculated setting the PLY in the supernatant of D39 to the value of 1. The MOI of 20 used for D39 infection of neuronal cells was then multiplied by the coefficient factors of 0.75, 0.61 and 0.63 to match the same amount of released PLY for the infection experiments with, respectively, the serotypes 6A, 15A and 16F pneumococcal meningitis clinical isolates.

**Meningitis clinical isolate**	**PLY in supernatant compared to the PLY in supernatant of D39** (PLY/GADH ratio of clinical isolate divided by PLY/GAPDH ratio of D39)	**Coefficient factor to match the released PLY of D39** (PLY in supernatant of D39 set to 1)
Serotype 6A	1.33	1/1.33 = 0.75
Serotype 15A	1.64	1/1.64 = 0.61
Serotype 16F	1.58	1/1.58 = 0.63
